# The impact of obesity on different glucose tolerance status with incident cardiovascular disease and mortality events over 15 years of follow-up: a pooled cohort analysis

**DOI:** 10.1186/s13098-023-01253-0

**Published:** 2024-01-25

**Authors:** Samaneh Asgari, Danial Molavizadeh, Kiarash soltani, Davood Khalili, Fereidoun Azizi, Farzad Hadaegh

**Affiliations:** 1 Prevention of Metabolic Disorders Research Center, Research Institute for Endocrine Sciences, Shahid Beheshti University of Medical Sciences, 19395-4763, Tehran, Islamic Republic of Iran; 2 School of Medicine, Kashan University of Medical Sciences, Kashan, Iran; 3 School of Medicine, Shahid Beheshti University of Medical Sciences, Tehran, Iran; 4 Endocrine Research Center, Research Institute for Endocrine Sciences, Shahid Beheshti University of Medical Sciences, Tehran, Iran

**Keywords:** Obesity, Glucose intolerance status, Cardiovascular disease, Mortality

## Abstract

**Background:**

The effect of obesity in different glucose tolerance statuses i.e. normoglycemia (NGT), pre-diabetes, and type 2 diabetes (T2DM) on cardiovascular disease (CVD) and mortality has been an area of ongoing debate and uncertainty. In the present study, we aimed to examine the impact of being obese, whether general or central separately, in comparison with non-obese in different glucose tolerance statuses on the above outcomes.

**Methods:**

The study population included 18,184 participants aged 30–60 years (9927 women) from three longitudinal studies, including Atherosclerosis Risk in Communities, Multi-Ethnic Study of Atherosclerosis, and Tehran Lipid and Glucose Study. Glucose tolerance status was defined as NGT (fasting plasma glucose < 5.55 mmol/L), pre-diabetes (5.55–7.00 mmol/L), and T2DM (≥ 7 mmol/L or taking any medication for diabetes). Moreover, general and central obesity were defined based on body mass index and waist circumference (WC), respectively. Multivariable stratified Cox regression analysis was used to estimate hazard ratios (HRs (95% CI)) for CVD and mortality events.

**Results:**

During a 16-year follow-up, 2733 CVD events, 1101 CV mortality, and 3678 all-cause mortality events were recorded. We observed that being generally obese in comparison with non-obese increased the risk of CV and all-cause mortality in all glucose tolerance statuses; while considering CVD events, only among individuals with T2DM, the presence of general obesity was associated with marginally significant higher risk [1.19 (0.98–1.43); p-value = 0.07]. Regarding central adiposity, multivariate analysis revealed that elevated WC in NGT participants is associated with incident CVD [1.27(1.12–1.46)] and all-cause mortality [1.13(1.00–1.28)]. Moreover, central adiposity increased the risk of CV mortality in pre-diabetes individuals [1.47 (1.11–1.95)].

**Conclusion:**

Findings from this pooled prospective cohort studies provide evidence that general obesity shows an unfavorable association with CV and all-cause mortality among the general population irrespective of their glucose tolerance statusThe findings imply that it's important to take into account the requirement and magnitude of weight reduction in people who are obese when offering guidance.

**Supplementary Information:**

The online version contains supplementary material available at 10.1186/s13098-023-01253-0.

## Background

Type 2 diabetes mellitus (T2DM) and pre-diabetes are well-established and independent risk factors for various life-threatening conditions, particularly cardiovascular diseases and mortality [1, 2]. Over the past few decades, the global prevalence and incidence of pre-diabetes/T2DM have been steadily increasing, raising significant concerns. According to the International Diabetes Federation (IDF), the worldwide prevalence of prediabetes and T2DM was 6.2% and 10.5% in 2021 and estimated reach to 6.9% and 12.2% in 2045, respectively [3]. The meta-analysis of Mendelian randomization studies suggests that obesity was associated with 67% and 20% higher risk of T2DM and coronary artery disease (CAD), respectively with significant heterogeneity between included studies [4].

Recently, in a retrospective study among the American population, researcher found that among obese individuals those with pre-diabetes (using A1C criteria) had a higher risk of mortality compared to the normoglycemic population [5]. However, a post hoc analysis of the ORIGIN study found that overweight and moderately obese patients had a lower risk of all-cause mortality compared to patients with a BMI of 22–24.9 kg/m^2^ among both diabetic and prediabetic populations [6]. Moreover, some recent studies have investigated sex-specific differences in CVD incidence, CV mortality, and all-cause mortality, taking into account obesity status [7–9]. While some studies have found obesity to be a protective factor in women [7], others have reported contradictory results [10].

The combined impact of obesity, and diabetes on cardiovascular disease (CVD) and all-cause mortality has been an area of ongoing debate and uncertainty [11–13]. In 1973, Sims et al. [14] introduced the term “diabesity” to describe the synergistic increase in the risk of cardiometabolic disorders when diabetes and obesity coexist. Among the rural Chinese population, it was shown that obesity decreased the risk of all-cause mortality among the diabetes population [15]. Moreover, in another study, no significant association was observed in prediabetic individuals who were also obese [16].

Given the potential impact of ethnicity, obesity patterns, and lifestyle on public health recommendations, it has becomes important to examine the association of obesity, whether as central or general, for incident CVD, CV, and total in different glucose tolerance statuses for incident CVD, CV, and all-cause mortality, as well as the association between diabesity phenotypes and outcomes. Conducting a pooled analysis using data from three cohorts (ARIC, MESA, and TLGS) can provide valuable insights into this stratification. We also aimed to investigate the sex-specific risks of CVD, CV mortality, and all-cause mortality for each diabesity phenotype in the pooled cohort dataset.

## Methods

### Study population

The current research involved analyzing data from three large population-based studiesincluding the Atherosclerosis Risk in Communities (ARIC) study, the Multi-Ethnic Study of Atherosclerosis (MESA), and the Tehran Lipid and Glucose Study (TLGS). The ARIC and MESA datasets were obtained from the National Heart, Lung, and Blood Institute's Biologic Specimen and Data Repository Information Coordinating Center (BioLINCC). The details regarding the study designs, objectives, and participant characteristics for each cohort have been previously published [17–20] and are briefly summarized in the Additional file 1.

For the present analysis, the pooled and harmonized individual-level data of a total of 20,882 individuals aged 30–60 years from the initial phases of ARIC (1987–89; n = 12,162), MESA (2000–02; n = 3100), and phase two for TLGS (2002–05; n = 5560) cohorts were considered as the baseline population. Exclusions were carried out at two separate lines for mortality and CVD event analysis. For mortality-related files, among 20,882 participants (11,476 women) from the baseline examination, after exclusions of 2654 cases with missing data on any examined baseline variables and 44 participants with no follow-up from baseline examination; 18,184 participants (9927 women) remained (mean follow-up of 18.8 years, response rate 87%). For CVD event analysis, 20,882 participants aged 30–60 years or more were selected. Subjects with prevalent CVD on baseline examination (N = 942), missing data (N = 2467), or without any follow-up (N = 40) were excluded, leaving 17,433 participants (9,682 women) for the current study (mean follow-up of 16.6 years, response rate 87%) (Additional file 1: Fig S1).

### Definition of variables

Variable definitions were harmonized across the three cohorts according to the previously published articles [21–25] as shown in Box 1. A further description of the study cohort and local covariates is presented in the Additional file 1.The ethnicity-specific cut-off point for WC was considered [26] and there were minor differences in the definition of the prevalence of CVD among cohorts that were applied.

**Table:** Box1 Definition of different cardiovascular risk factors categories, pooled cohort data

variables	Definitions
Glucose tolerance categories	
- Normoglycemia	FPG < 5.55 mmol/Land taking no anti-diabetic medications
- Pre-diabetes	5.55 mmol/L ≤ FPG < 7 mmol/L and taking no anti-diabetic medications
- Type 2 diabetes	FPG ≥ 7 mmol/l or taking any medication for diabetes
General obesity, yes	BMI ≥ 30 kg/m^2^
Central adiposity, yes	**For TLGS:** WC ≥ 95 cm for Iranian men and women [46] **For ARIC and MESA:** WC ≥ 88 cm for American men and ≥ 102 cm for American women
Hypertension, yes	SBP ≥ 140 mmHg or DBP ≥ 90 mmHg or taking any medication for hypertension
Non-HDL-C	Total cholesterol-HDL-C
Family history of CVD, yes	History of myocardial infarction, stroke, or sudden cardiac death in a men first-degree relative < 55 years or in a women first-degree relative < 65
Prevalent CVD, yes	**For TLGS:** history of acute coronary syndrome leading to CCU admission, history of percutaneous coronary intervention (PCI), or coronary artery bypass graft (CABG), angiographic proven coronary artery disease (i.e., > 50% luminal narrowing of one or more coronary artery), or history of stroke events **For ARIC and MESA:** History of heart failure (HF), myocardial infarction (MI), coronary revascularization, stroke, or transient ischemic Attack
Current smoking, yes	Participants who used any tobacco product (cigarette, pipe, and water pipe) at the time of examination
Education, years	
− < 6 (Reference)	Illiterate participants and those with primary school education or less (less than 6 years of education)
− 6–12	Those who had a diploma or did not complete the diploma but finished primary school (6 years)
− ≥ 12	Those with higher than a diploma education (more than 12 years of education)
Diabesity phenotype	
− 1(Reference)	NGT & non-obese
− 2	NGT& obese
− 3	Pre-diabetes& non-obese
− 4	Pre-diabetes & obese
− 5	T2DM& non-obese
− 6	T2DM& obese

FPG fasting plasma glucose, SBP systolic blood pressure, DBP diastolic blood pressure, BMI body mass index, WC waist circumference, FPG fasting plasma glucose, non-HDL-C non-high-density lipoprotein cholesterol, FHCVD family history cardiovascular disease, CVD cardiovascular disease, NGT normal glucose tolerance.

### Outcomes

#### ARIC

Incident CVD was described as a combination of new cases of CHD, stroke, or heart failure (HF) occurring between the initial assessment and December 31, 2015 (median follow-up = 25.5 years). Throughout the study period, participants were continuously monitored for any potential CVD events through yearly telephone inquiries, monitoring of local hospitals, and cross-referencing with state and national death records. An expert panel reviewed and confirmed all instances of CHD and stroke. Incident CHD was identified as a confirmed or likely non-fatal heart attack or confirmed fatal CHD. The stroke was categorized as a confirmed or likely ischemic or hemorrhagic stroke. HF was determined by the first hospital admission or death linked to HF, based on the utilization of ICD-9 code 428 or ICD-10 code I-50 during the primary analyses. Further information regarding CVD surveillance in ARIC has been previously documented [23]. The researchers determined how the study participants death by regularly checking up on them, monitoring hospitals in the community, and cross-referencing with local and national death records. The accuracy of the date and cause of death was confirmed by reviewing the death certificates. All-cause mortality referred to deaths from any cause, while cardiovascular (CV) mortality referred to deaths where the main cause was related to cardiovascular diseases, using specific medical codes (ICD-9 codes 390–459 or ICD-10 codes I00-I99) [20].

#### MESA

In this scientific study, the participants were monitored for the occurrence of new cardiovascular events and mortality at intervals of 9 to 12 months. A telephone interviewer contacted each participant to gather information about any hospital admissions, outpatient diagnoses, and procedures related to cardiovascular issues, as well as deaths that occurred between the follow-up intervals. Additionally, the study occasionally discovered additional events through clinic visits, participant reports, medical record reviews, or obituaries. To ensure the accuracy of self-reported diagnoses, the study requested copies of death certificates and medical records for all hospitalizations and selected outpatient cardiovascular diagnoses and procedures. In cases of cardiovascular deaths that occurred outside of a hospital setting, interviews were conducted with the next of kin. The study was successful in obtaining hospital records for approximately 99% of suspected cases of hospitalized cardiovascular events. A panel of physicians centrally reviewed and confirmed the occurrence of cardiovascular events for the analysis. In this study, CVD was defined as a combination of myocardial infarction, angina, resuscitated cardiac arrest, stroke (excluding transient ischemic attack), death due to CHD, stroke-related death, death from other atherosclerotic causes, or other cardiovascular disease-related death. The cause of death was determined through committee review for potential CVD-related deaths, while the underlying cause of death for other cases was obtained from state or city vital statistics departments [21].

#### TLGS

TLGS outcome data was collected previously in a specific manner. Trained nurses conducted annual telephone follow-ups with participants to ask about any new medical events. Trained physicians then gathered additional information through home visits or by reviewing medical records related to the reported events. An outcome committee, consisting of experts from various medical fields, assessed the data and assigned specific outcome codes to each event. In this study, the focus was on coronary heart disease (CHD) events as outcomes. These events included cases of definite myocardial infarction (MI) diagnosed using electrocardiogram (ECG) results and biomarkers. Probable MI cases were identified by positive ECG findings along with cardiac symptoms or signs, and biomarker results that were inconclusive or negative. Unstable angina pectoris was characterized by new cardiac symptoms or changing patterns of symptoms, accompanied by positive ECG findings and normal biomarker levels. Angiography-proven CHD and CHD-related deaths, including deaths occurring in the hospital due to CHD and sudden cardiac deaths caused by cardiac disease within one hour of symptom onset, were also considered CHD events based on specific criteria or verbal autopsy documents.

Stroke was categorized into three groups: definite stroke, possible stroke, and transient ischemic attack (TIA). Definite stroke follows the World Health Organization (WHO) definition, involving the rapid onset of clinical signs or global disturbance of cerebral function lasting more than 24 h or resulting in death with no apparent cause other than of vascular origin. Imaging results indicating stroke and acute brain injuries in patients with symptoms rapidly disappearing were also considered. Possible stroke included cases where there was an acute focal neurological deficit without imaging evidence of stroke or when the data did not fully meet the WHO definition of definite stroke. TIA cases were identified when symptoms resolved within 24 h.

Cardiovascular disease (CVD) was defined as a combination of CHD events, stroke, or death due to cerebrovascular causes. In cases of mortality, data were collected either from hospitals by authorized local physicians or from death certificates and were subsequently evaluated by the TLGS outcome committee. The study also examined a composite outcome that included both CVD events and events related to all-cause mortality [27].

### Statistical analysis

The baseline characteristics of the participants were described using mean (standard deviation; SD) for continuous variables, while numbers and proportions (%) were used for categorical variables, based on different combinations of glucose tolerance statuses and general obesity in men and women, separately. Statistical analyses were conducted to compare continuous variables using ANOVA and t-tests, as deemed suitable. The chi-square test was employed to compare categorical variables. To determine the incidence rates, crude rates per 1000 person-years along with their corresponding 95% confidence intervals (95% CI) were calculated for each outcome.

Multivariable Cox regression analysis was utilized to evaluate how general obesity (defined by BMI), and central adiposity (defined by WC) impact the risk of CVD, CV mortality, and all-cause mortality. A literature review was conducted to consider conventional covariates such as age, sex (for the overall population), education levels, current smoking, hypertension, FH-CV, non-HDL-C, and prevalent CVD (except for CVD) in our data analysis [21–23, 28]. Additionally, we calculated the hazard ratios (HRs) and 95% confidence intervals (CIs) to assess the combined association of obesity and diabetes status on the occurrence of specified outcomes. Notably, no significant gender interactions were observed (the minimum interaction p-value of 0.045 exceeded the Bonferroni-corrected significance level of 0.003). However, all analyses were conducted separately for men, women, and the total population.

To address potential variations in baseline hazard risk, population characteristics, or data collection methods among the cohort studies, cohort-stratified baseline hazards were employed to address the study-to-study heterogeneity [29, 30]. The Cox model was utilized to evaluate the proportional hazards assumption, and the Schoenfeld residual test was employed for this purpose. The assessment revealed that all proportionality assumptions were met and considered appropriate. All statistical analyses were performed using STATA version 17 and 2-sided P values < 0.05 were considered statistically significant.

## Results

### Baseline characteristics

The study population included 18,184 participants (9927 women) with a mean (SD) age of 50.0 (7.1) years. The baseline characteristics of individuals in each cohort study stratified by gender are summarized in the Additional file 1: Tables S1. Compared to normal glucose tolerance and non-obese (NGT & non-obese), participants with both obesity and diabetes tended to be older and had a higher likelihood of having poor metabolic conditions, including elevated SBP and DBP, WC, BMI, Non-HDL-C, and FPG. Additionally, conditions such as hypertension, CVD, and FH-CVD were more prevalent among diabetic and obese men, except for a current smoker, as shown in Table 1. Similar patterns were observed among women, where individuals with diabetes & obesity, compared to NGT & non-obese women, tended to be older with poor metabolic conditions. Furthermore, similar to men, hypertension, CVD, and FH-CVD were more common among women with diabetes & obese phenotype, except for smoking (Table 2).

**Table 1:** Baseline characteristics of subjects regarding different combinations of glucose tolerance statuses and general obesity among men

Variables	NGT		Pre-diabetes		Type 2 diabetes	
	Non-obese (n = 3918)	Obese (n = 1057)	Non-obese (n = 1828)	Obese (n = 736)	Non-obese (n = 379)	Obese (n = 339)
Age (years)	49.0 (7.6)	49.6 (7.4)	51.9 (5.6)	51.2 (5.8)	52.7 (6.0)	52.6 (5.7)
SBP (mmHg)	115.0 (15.8)	122.2 (18.1)	120.9 (17.4)	124.9 (16.1)	126.2 (20.9)	129.4 (18.5)
DBP (mmHg)	72.9 (10.7)	75.5 (11.5)	75.4 (11.2)	78.1 (11.1)	76.8 (12.5)	77.0 (11.3)
BMI (kg/m^2^)	25.2 (2.8)	33.8 (4.0)	26.1 (2.4)	33.7 (4.1)	26.2 (2.6)	34.7 (4.1)
WC (cm)	90.8 (9.1)	109.6 (10.5)	95.6 (7.4)	112.6 (10.6)	95.7 (7.7)	113.9 (10.4)
Non-HDL-C (mg/dl)	151.0 (126.0–178.4)	153.0 (130.5–178.3)	165.4 (141.0–191.6)	169.0 (142.6–192.6)	168.0 (140.1–193.4)	160.1 (135.0–196.2)
FPG (mg/dl)	90.0 (84.0–95.0)	90.0 (85.0–95.0)	106.0 (102.1–111.0)	106.9 (103.0–113.3)	150.0 (130.0–209.0)	154.0 (132.0–205.1)
Education, years						
< 6	217 (5.5)	45 (4.3)	51 (2.8)	27 (3.7)	28 (7.4)	17 (5.0)
6–12	1105 (28.2)	249 (23.5)	440 (24.1)	165 (22.4)	141 (37.2)	70 (20.7)
≥ 12	2596 (66.3)	763 (72.2)	1337 (73.1)	544 (73.9)	210 (55.4)	252 (74.3)
Current smoker (yes)	1190 (30.4)	262 (24.8)	531 (29.1)	179 (24.3)	118 (31.1)	63 (18.6)
Hypertension (yes)	727 (18.6)	364 (34.4)	561 (30.7)	323 (43.9)	159 (41.9)	214 (63.1)
FH-CVD (yes)	718 (18.3)	301 (28.5)	195 (10.7)	119 (16.2)	58 (15.3)	80 (23.6)
Prevalent CVD (yes)	178 (4.5)	40 (3.8)	132 (7.2)	62 (8.4)	49 (12.9)	45 (13.3)

Continuous variables are shown as mean (standard deviation; SD) for normal distribution and median (interquartile range: IQR) for skewed variables (e.g. FPG, non-HDL-C), and categorical variables are presented as number (%)

ARIC: Atherosclerosis Risk in Communities; MESA: Multi-Ethnic Study of Atherosclerosis; TLGS: Tehran Lipid and Glucose Study; FPG: fasting plasma glucose; SBP: systolic blood pressure; DBP: diastolic blood pressure; BMI: body mass index; WC: waist circumference; FPG: fasting plasma glucose; non-HDL-C: non-high-density lipoprotein cholesterol; FHCVD: family history cardiovascular disease; CVD: cardiovascular disease; normal glucose tolerance (NGT). General obesity (BMI ≥ 30 kg/m^2^)

Normoglycemia: FPG < 100 mg/dl & no medication; pre-diabetes: FPG 100–126 mg/dl and no medication; type 2 diabetes; FPG ≥ 126 mg/dl or using medication

**Table 2:** Baseline characteristics of subjects regarding different combinations of glucose tolerance statuses and general obesity among women

Variables	NGT		Pre-diabetes		Type 2 diabetes	
	Non-obese (n = 4960)	Obese (n = 1722)	Non-obese (n = 1379)	Obese (n = 950)	Non-obese (n = 384)	Obese (n = 532)
Age (years)	48.7(7.5)	48.5(7.3)	51.9(5.5)	51.3(5.9)	52.9(5.8)	52.3(5.3)
SBP (mmHg)	112.3(16.1)	120.1(17.4)	119.3(18.7)	124.9(17.7)	125.4(20.6)	130.4(20.2)
DBP (mmHg)	71.0(10.0)	76.8(10.1)	73.3(10.3)	77.4(10.1)	74.8(10.2)	77.7(11.0)
BMI (kg/m^2^)	24.9(2.9)	33.8(3.6)	25.6(2.8)	35.1(4.6)	26.5(2.5)	35.6(4.7)
WC (cm)	86.4(9.4)	105.6(11.2)	89.7(9.4)	110.5(12.3)	93.7(8.3)	113.8(12.3)
Non-HDL-C (mg/dl)	144.0(120.1–172.0)	156.0(183.0–132.0)	159.0(133.0–189.2)	165.1(136.7–193.0)	168.0(139.0–201.0)	171.0(142.0–205.0)
FPG (mg/dl)	89.6(85.0–94.0)	90.7(86.0–95.0)	105.0(102.0–109.0)	107.0(103.0–112.7)	157.0(130.0–218.0)	165.0(134.0–241.5)
Education, years						
< 6	343(6.9)	287(16.7)	75(5.4)	131(13.8)	76(19.8)	102(19.2)
6–12	1231(24.8)	586(34.0)	270(19.6)	292(30.7)	97(25.3)	161(30.3)
≥ 12	3386(68.3)	849(49.3)	1034(75.0)	527(55.5)	211(54.9)	269(50.5)
Current smoker(yes)	1021(20.6)	225(13.1)	352(25.5)	142(14.9)	76(19.8)	98(18.4)
Hypertension (yes)	940(19.0)	549(31.9)	489(35.5)	480(50.5)	191(49.7)	332(62.4)
FH-CVD (yes)	746(15.0)	255(14.8)	170(12.3)	1234(12.9)	68(17.7)	93(17.5)
Prevalent CVD (yes)	65(1.3)	36(2.1)	40(2.9)	40(4.2)	21(5.5)	43(8.1)

Continuous variables are shown as mean (standard deviation; SD) for normal distribution and median (interquartile range: IQR) for skewed variables (e.g. FPG, non-HDL-C), and categorical variables are presented as number (%)

*ARIC* Atherosclerosis Risk in Communities, *MESA* Multi-Ethnic Study of Atherosclerosis, *TLGS* Tehran Lipid and Glucose Study, *FPG* fasting plasma glucose, *SBP* systolic blood pressure, *DBP* diastolic blood pressure, *BMI* body mass index, *WC* waist circumference, *FPG* fasting plasma glucose, *non-HDL-C* non-high-density lipoprotein cholesterol, *FHCVD* family history cardiovascular disease, *CVD* cardiovascular disease, *NGT* normal glucose tolerance. General obesity (BMI ≥ 30 kg/m^2^)

Normoglycemia: FPG < 100 mg/dl & no medication; pre-diabetes: FPG 100–126 mg/dl and no medication; type 2 diabetes; FPG ≥ 126 mg/dl or using medication

### Incident CVD

Over a median follow-up period of 16.3 years (interquartile range (IQR): 8.8–25.3 years), 1172 women and 1561 men were diagnosed with CVD. The annual CVD incidence rate for the entire population was 9.4 cases per 1000 person-years (95% CI 9.1–9.8). When we break it down by gender, the incidence rates were 13.1 per 1000 person-years (95% CI 12.5–13.7) for men and 6.9 per 1000 person-years (95% CI 6.5–7.3) for women. The annual incidence rate of CVD defined by different categories of diabetes and obesity is presented in the Additional file 1: Fig S2.

**Fig. 1:**
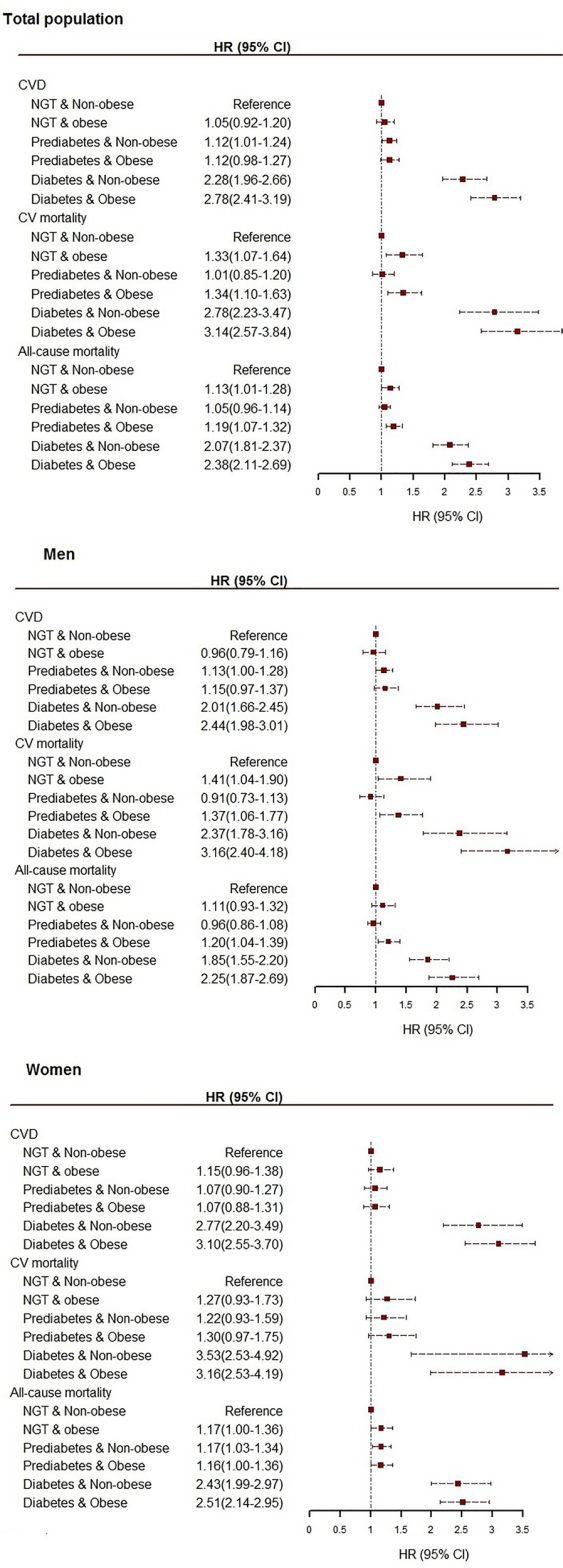
Multivariable adjusted HRs (95% CI) for incident CVD, CV mortality, and all-cause mortality with diabesity phenotypes in the total population, men, and women. General obesity (BMI ≥ 30 kg/m^2^). Model adjusted for sex (total population), age, non-HDL-C, education levels, hypertension, FH-CVD, and prevalent CVD (except for CVD). *HR* hazard ratio, *CI* confidence interval, *CVD* cardiovascular disease, *FHCVD* family history CVD, *HDL_C* high-density lipoprotein cholesterol, *BMI* body mass index, *NGT* normal glucose tolerance. NGT: FPG < 100 mg/dl & no medication; prediabetes: FPG 100-126 mg/dl and no medication; type 2 diabetes; FPG ≥ 126 mg/dl or using medication

Considering the diabesity definition (NGT & non-obese as reference), the pre-diabetes & non-obese [HR (95% CI 1.12 (1.01–1.24)], diabetes & non-obese [2.28 (1.96–2.66)], and diabetes & obese phenotypes [2.78 (2.41–3.19)] were associated with a higher risk of incident CVD in total population (Fig. 1). As shown in Fig. 1 in terms of diabesity, both men and women with diabetes & obesity had higher risks of developing CVD. The HR (95% CI) for CVD was 3.10 (2.55–3.70) for diabetic women with obesity, 2.77 (2.20–3.49) for diabetic women without obesity, 2.44 (1.98–3.01) for diabetic men with obesity, and 2.01 (1.66–2.45) for diabetic men without obesity.

Multivariable adjusted HRs (95% CI) of obesity for incident CVD in different glucose tolerance statuses among men, women, and the total population separately are shown in Table 3. However, no significant association was found in the total population, among NGT women, general obesity increased the risk of incident CVD by 24%, compared to non-obese counterparts while through diabetic men, obesity was associated with 32% excess risk of CVD incidence. Moreover, in the total population among the NGT group, central adiposity increased the risk of incident CVD by 27% and by 51% specifically in women (Table 4).

**Table 3:** Multivariable adjusted HRs (95% CI) of general obesity for incident CVD, CV, and total mortality in different glucose tolerance statuses among men, women, and the total population separately

		NGT		Pre-diabetes		With T2DM	
		Non-Obese	Obese	Non-Obese	Obese	Non-Obese	Obese
CVD							
Women	E/N	424/4895	166/1686	190/1339	134/910	91/363	167/489
	HR (95% CI)	Reference	**1.24(1.03–1.50)**	Reference	1.05(0.83–1.32)	Reference	1.11(0.85–1.46)
	p-value		**0.02**		0.70		0.44
Men	E/N	549/3740	125/1017	474/1696	179/674	126/330	108/294
	HR (95% CI)	Reference	1.00(0.81–1.21)	Reference	1.07(0.90–1.27)	Reference	**1.32(1.01–1.73)**
	p-value		0.94		0.42		0.046
Total	E/N	973/8635	291/2703	664/3035	313/1584	217/693	275/783
	HR (95% CI)	Reference	1.07(0.93–1.22)	Reference	1.07(0.93–1.23)	Reference	1.19(0.98–1.43)
	p-value		0.33		0.34		0.07
CV mortality							
Women	E/N	144/4960	58/1722	86/1379	65/950	49/380	79/532
	HR (95% CI)	Reference	**1.60(1.15–2.20)**	Reference	1.27(0.91–1.79)	Reference	0.91(0.62–1.32)
	p-value		**0.005**		0.16		0.62
Men	E/N	174/3918	58/1057	159/1828	89/736	66/374	74/339
	HR (95% CI)	Reference	**1.41(1.04–1.92)**	Reference	**1.59(1.23–2.07)**	Reference	**1.48(1.04–2.12)**
	p-value		**0.03**		** < 0.001**		**0.03**
Total	E/N	318/8878	116/2779	245/3207	154/1686	115/754	153/871
	HR (95% CI)	Reference	**1.81(1.49–2.21)**	Reference	**1.52(1.23–1.89)**	Reference	**1.58(1.22–2.04)**
	p-value		** < 0.001**		** < 0.001**		** < 0.001**
All-cause mortality							
Women	E/N	653/4960	214/1722	335/1379	215/950	115/384	208/532
	HR (95% CI)	Reference	**1.38(1.17–1.63)**	Reference	1.14(0.95–1.37)	Reference	1.06(0.84–1.35)
	p-value		** < 0.001**		0.15		0.60
Men	E/N	652/3918	156/1057	572/1828	252/736	159/379	147/339
	HR (95% CI)	Reference	1.12(0.94–1.35)	Reference	**1.36(1.17–1.58)**	Reference	**1.30(1.02–1.65)**
	p-value		0.20		** < 0.001**		**0.03**
Total	E/N	1305/8878	370/2779	907/3207	467/1686	274/763	355/871
	HR (95% CI)	Reference	**1.52(1.38–1.68)**	Reference	**1.38(1.23–1.54)**	Reference	**1.35(1.14–1.60)**
	p-value		** < 0.001**		** < 0.001**		** < 0.001**

General obesity (BMI ≥ 30 kg/m^2^)

Model adjusted for gender (total population), age, non-HDL-C, current smoking, education levels, hypertension, FHCVD, and prevalent CVD (except for CVD). *E* number of events, *N* number of population, *HR* hazard ratio, *CI* confidence interval, *CVD* cardiovascular disease, *FHCVD* family history CVD, *HDL_c* high-density lipoprotein cholesterol, *BMI* body mass index, *NGT* normal glucose tolerance

Normoglycemia: FPG < 100 mg/dl & no medication; pre-diabetes: FPG 100–126 mg/dl and no medication; type 2 diabetes; FPG ≥ 126 mg/dl or using medication

Significant values are bolded

**Table 4:** Multivariable adjusted HRs (95% CI) of central adiposity for incident CVD, CV, and total mortality in different glucose tolerance statuses among men, women, and the total population separately

		NGT		Pre-diabetes		With T2DM	
		Non-obese	Obese	Non-obese	Obese	Non-obese	Obese
CVD							
Women	E/N	401/4870	189/1711	189/1324	135/925	79/311	179/541
	HR (95% CI)	Reference	**1.51(1.19–1.94)**	Reference	0.79(0.57–1.09)	Reference	0.98(0.70–1.39)
	p-value		**0.001**		0.15		0.94
Men	E/N	136/1680	538/3077	64/313	589/2057	18/73	216/551
	HR (95% CI)	Reference	1.10(0.89–1.39)	Reference	0.97(0.73–1.30)	Reference	1.10(0.64–1.89)
	p-value		0.43		0.85		0.72
Total	E/N	537/6550	727/4788	253/1637	724/2982	97/384	395/1092
	HR (95% CI)	Reference	**1.27(1.12–1.46)**	Reference	1.03(0.87–1.22)	Reference	1.13(0.89–1.43)
	p-value		** < 0.001**		0.72		0.29
CV mortality							
Women	E/N	143/4933	59/1749	81/1365	70/964	39/325	89/591
	HR (95% CI)	Reference	1.36(0.98–1.87)	Reference	**1.42(1.02–2.00)**	Reference	1.05(0.71–1.55)
	p-value		0.06		**0.04**		0.79
Men	E/N	39/1728	193/3247	13/331	235/2233	9/88	131/630
	HR (95% CI)	Reference	1.12(0.78–1.62)	Reference	1.67(0.94–2.94)	Reference	1.09(0.55–2.21)
	p-value		0.54		0.08		0.79
Total	E/N	182/6661	252/4996	94/1696	305/3197	48/413	220/1221
	HR (95% CI)	Reference	1.22(0.96–1.56)	Reference	**1.47(1.11–1.95)**	Reference	1.08(0.77–1.51)
	p-value		0.1		**0.01**		0.65
All-cause mortality							
Women	E/N	649/4933	218/1749	333/1356	217/964	97/325	226/591
	HR (95% CI)	Reference	**1.24(1.06–1.46)**	Reference	1.09(0.91–1.31)	Reference	1.13(0.89–1.44)
	p-value		**0.01**		0.31		0.31
Men	E/N	154/1728	654/3247	71/331	753/2233	26/88	280/630
	HR (95% CI)	Reference	1.03(0.85–1.24)	Reference	1.06(0.83–1.37)	Reference	0.77(0.51–1.17)
	p-value		0.78		0.63		0.23
Total	E/N	803/6661	872/4996	404/1696	970/3197	123/413	506/1221
	HR (95% CI)	Reference	**1.13(1.00–1.28)**	Reference	1.08(0.93–1.25)	Reference	1.06(0.86–1.31)
	p-value		**0.04**		0.29		0.59

Central adiposity (WC ≥ 95 cm for Iranian men and women; ≥ 88 cm for American men and ≥ 102 cm for American women)

Model adjusted for gender (total population), age, non-HDL-C, current smoking, education levels, hypertension, FHCVD, and prevalent CVD (except for CVD). *E* number of events, *N* number of population, *HR* hazard ratio, *CI* confidence interval, *CVD* cardiovascular disease, *FHCVD* family history CVD, *HDL_c* high-density lipoprotein cholesterol, *BMI* body mass index, *NGT* normal glucose tolerance

Normoglycemia: FPG < 100 mg/dl & no medication; pre-diabetes: FPG 100–126 mg/dl and no medication; type 2 diabetes; FPG ≥ 126 mg/dl or using medication

Significant values are bolded

### CV mortality

During the median (IQR) follow-up of 16.7 years (11.2–25.7), there were 1101 CV mortalities, with 481 of them occurring in women. The annual crude CV mortality rates in the overall population were 3.4 per 1000 person-years (95% CI 3.2–3.6). When analyzed by sex, the rates were 4.4 per 1000 person-years (95% CI 4.1–4.7) in men and 2.6 per 1000 person-years (95% CI 2.4–2.9) in women. The annual incidence rate of CV mortality defined by different categories of diabesity phenotypes is presented in the Additional file 1: Fig S3.

Evaluating different diabesity phenotypes, defined by BMI, in the total population, revealed that, compared to NGT & non-obese individuals, phenotypes accompanying obesity or diabetes, were positively associated with CV mortality: NGT & obese [1.33 (1.07–1.64)], prediabetes & obese [1.34 (1.10–1.63)], diabetes & non-obese [2.78 (2.23–3.47)], diabetes & obese [3.14 (2.57–3.84)] (Fig. 1). Similarly, in sex-stratified analysis, diabetes & obesity were associated with an increased risk of CV mortality in men (Fig. 1); however; in women, diabetes irrespective of obesity status, increased the risk of CV mortality (Fig. 1). As shown in Table 3, general obesity increased the risk of CV mortality in the total population in NGT [1.81(1.49–2.21)], pre-diabetes [1.52(1.23–1.89)], and diabetes [1.58(1.22–2.04)] status; generally, the associations were similar to those of the men population. Additionally, as shown in Table 4, central adiposity increased the risk of CV mortality in the total population in pre-diabetes [1.47(1.11–1.95)]; generally, the associations were similar to those of the women.

### All-cause mortality

Over a median follow-up period of 16.7 years (IQR: 11.2–25.7), there were a total of 3678 all-cause deaths, with 1740 of them occurring in women. The annual crude mortality rates in the overall population were 11.3 per 1000 person-years (95% CI 11.0–11.7). When analyzed by sex, the rates were 13.7 per 1000 person-years (95% CI 13.1–14.3) in men and 9.5 per 1000 person-years (95% CI 9.1–9.9) in women. The annual incidence rate of all-cause mortality defined by different categories of diabesity phenotypes is presented in the Additional file 1: Fig S4.

Evaluating different diabesity phenotypes in the total population revealed that except prediabetes & non-obese, all other phenotypes were positively associated with all-cause mortality, and the highest HR was observed in diabetes & obesity (2.38; 2.11–2.96) (Fig. 1). based on sex-stratified analysis, prediabetes & obese (1.20; 1.04–1.39), diabetes & non-obese (1.85; 1.55–2.20), diabetes & obese (2.25; 1.87–2.69) men, have elevated risk for all-cause mortality (Fig. 1). Furthermore, in women, pre-diabetes & non-obese (1.17; 1.03–1.34), diabetes & non-obese (2.43; 1.99–2.97), diabetes & obese (2.51; 2.14–2.95) were associated with all-cause mortality (Fig. 1). Nonetheless, the interaction term showed a lack of significance, suggesting that there is no statistically meaningful indication of a distinction between men and women concerning the association between obesity, diabetes, and mortality. For the total population, mortality was higher in obese than in non-obese individuals (HR 1.24; 95% CI 1.15, 1.34).

Table 3 represents the multivariable-adjusted HRs (95% CI) of general obesity for all-cause mortality. When the analysis was done according to diabetes status, in the total population, general obesity was positively associated with all-cause mortality in NGT [1.52 (1.38–1.68)], prediabetic [1.38 (1.23–1.54)] and diabetic [1.35 (1.14–1.60)] subjects. Additionally, among men participants, obesity raised the risk of all-cause mortality in pre-diabetic [1.36 (1.17–1.58)] and diabetic [1.30 (1.02–1.65)] participants. However, in women, obesity significantly increased the risk of all-cause mortality only in NGT participants [1.38 (1.17–1.63)]. As shown in Table 4, it was noted that central adiposity was associated with an higher mortality rate in NGT participants [1.13 (1.00–1.28)]; this association was similarly identified in women [1.24 (1.06–1.46)].

## Discussion

In the present study, utilizing data from three large longitudinal studies with more than 18,000 participants, we aimed to investigate the additive impact of general and central obesity in different glucose tolerance statuses, for incident CVD and mortality events. Among the whole population, multivariate analysis showed that general obesity increased the risk of mortality events, in all of the glycemic states. Regarding central adiposity, multivariate analysis revealed that elevated WC in NGT participants is unfavorably associated with incident CVD and all-cause mortality, among the total population. Moreover, among pre-diabetic participants central adiposity exhibited an increased risk of CV mortality. Importantly, the impact of central adiposity was more prominent among women rather than men.

Harmonized individual-level data from 112 cohort studies conducted in 34 countries and 8 geographic regions showed that among men 4.3% and 10.2% of cases of incident CVD may be attributable to obesity and diabetes, respectively; the corresponding values for women are 3.6% and 15.2%, respectively [28]. While the independent impact of obesity, pre-diabetes, and T2DM in isolation on incident CVD and mortality has been widely reported [5, 31, 32], the joint effect of obesity status with different glucose tolerance is less addressed [16, 32]. In line with our findings in the context of diabesity phenotypes, a prospective cohort study among Chinese adults [16] demonstrated that in comparison to NGT & none-obese, phenotypes including diabetes, with or without obesity, was associated with 99% and 63% increased risk of developing CVD, respectively. They also reported that in the group of NGT participants, those who had general obesity exhibited a 92% higher risk of CVD. In another study within the UK Biobank involving 451,355 participants, it was reported that in comparison with normal BMI without diabetes category, the presence of general obesity in non-diabetic individuals was associated with a 35% elevated risk of CVD; this was about 88% among individuals with T2DM who had normal BMI range. In line with our findings, they also showed that the combined effect of both obesity and diabetes demonstrated a more than 2.5-fold higher risk of incident CVD [32]. Additionally, in our study, the phenotype of pre-diabetes without general obesity conferred an excess risk of incident CVD which is in line with a meta-analysis of 129 studies including 10 069 955 individuals suggesting pre-diabetes was associated with a 15% increased risk of CVD in a median follow-up time of 10 years [33]. Among the whole population, in terms of the added value of obesity in different glucose states status (Tables 3 and 4) for CVD events, we observed central adiposity among the NGT population was associated with about 30% higher risk; however, the addition of general obesity did not significantly increase the risk in different states of glucose tolerance. In line with our results, Sone et al. [34] indicated that excess WC (> 85 in women and > 90 in men) is not associated with incident CVD, either in men or women.

Considering CV mortality as an outcome, our data analysis showed that among all of the glycemic states, individuals who were generally obese had a higher risk of CV mortality, compared to their non-obese counterparts. In contrast to our findings, Doehner et al. [6] have studied the impact of BMI categories on CV mortality, among 12521 participants with T2DM or pre-diabetes, from ORIGIN trials. After 6.2 years of follow-up, they indicated that compared to subjects with a BMI of 22.24.9 kg/m2, obese participants showed about 30% lower risk of CV mortality. Furthermore, in a dose–response meta-analysis including 13 cohorts with 161,984 participants with T2DM, no significant association was found between obesity and the risk of cardiovascular mortality [35]. A systematic review and a dose–response analysis of 21 studies among 1, 349, 075 patients with type 2 diabetes reported that a BMI cut-off point > 28.4 kg/m^2^ was associated with a 12% higher risk for CV mortality without any significant gender difference [36]. However, in our data analysis, the unfavorable impact of general obesity among T2DM patients for CV mortality events was mainly observed only among men. In the context of central adiposity, we observed that elevated WC among pre-diabetic participants is associated with an excess risk of CV mortality.

The association between obesity, and all-cause mortality has been extensively studied in several studies [37–39], however, complete prognostic effects of obesity among different glycemic profiles are still inconclusive. The global population-attributable fraction of the 10-year risk of all-cause mortality for general obesity and T2DM was 2.9% and 12.2% for women, and 0.8% and 9.9% for men, respectively [28]. A large prospective cohort of 12.8 million Korean adults demonstrated that BMI ≥ 30 kg/m2 increases the risk of all-cause mortality in the NGT group by 8%; however, an inverse association was observed among those with newly diagnosed T2DM or prevalent diabetes [40]; moreover, the researchers also found that among IFG population, the general obesity was associated with all-cause mortality events only among women (HR (95% CI 1.06(1.02–1.10)). In contrast in our data analysis, the unfavorable impact of general obesity for all-cause mortality events among the IFG population was highlighted only among men. In line with our findings, Zahir et al. [41] in 2019 in an AusDiab study found that general obesity was associated with higher all-cause mortality and there was no evidence of the survival advantage of obesity in diabetic individuals. In another study, the results of a UK Biobank cohort study, diabetes with and without general obesity increases the risk of all-cause mortality to the same level (HR: 1.61 vs 1.57, respectively). They also reported that general obesity without diabetes also shows a significant association with all-cause mortality in comparison with non-diabetic participants with a BMI range of 18–25 kg/m^2^ [32]. We also found that having central obesity only among the NGT population increased the risk of all-cause mortality events.

Therefore our results can provide evidence for weight control irrespective of glucose tolerance status with considering lifestyle, nutritional, or pharmacological interventions for reducing the risk of mortality events. Weight loss benefits via lifestyle and nutritional interventions were supported by Look the Action for Health in Diabetes (Look AHEAD) randomized clinical trial [42, 43]. Moreover, Obesity pharmacotherapy as an adjunct to lifestyle interventions is another effective suggested method for those with BMI ≥ 27 kg/m^2^ or T2DM patients [44, 45].

The current study has several important strengths including pooled data cohort, long follow-up data for assessment of CVD and mortality, statistical power to check the association according to diabetes and obesity categories as well as gender stratified analysis. However, our study should be interpreted in the context of several limitations. First, the values of glycated hemoglobin A1C and 2-h post-load glucose were not available for all three cohorts, which may led to misclassifications in glycemic status. Second, despite we adjusted our analyses for a wide range of covariates, some residual unavoidable confounding such as socioeconomic, nutritional status, and duration of diabetes may affect the results. Finally, we could not investigate cause-specific mortality analysis due to the lack of data.

## Conclusion

In summary, findings from this pooled prospective cohort studies provide evidence that general obesity shows an unfavorable association with CV and all-cause mortality among the general population irrespective of their glucose tolerance status. However, the impact of central adiposity was found among NGT individuals for CVD and all-cause mortality events. The findings imply that it's important to take into account the requirement and magnitude of weight reduction in people who are obese when offering guidance.

### Supplementary Information

**Additional file 1: Table S1.** Baseline characteristics of subjects, stratified by study and gender. **Fig S1**. Study Flowchart *CV* Cardiovascular, *ARIC* Atherosclerosis Risk in Communities, *MESA* Multi-Ethnic Study of Atherosclerosis, *TLGS* Tehran Lipid and Glucose Study. **Fig S2.** The annual incidence rates of cardiovascular disease (CVD) by diabesity phenotypes in men and women, separately. Normoglycemia: FPG<100 mg/dl & no medication; pre-diabetes: FPG 100-126 mg/dl and no medication; type 2 diabetes; FPG ≥126 mg/dl or using medication. Obesity (BMI≥30 kg/m^2^). **Fig S3.** The annual incidence rates of cardiovascular (CV) mortality by diabesity phenotypes in men and women, separately. Normoglycemia: FPG<100 mg/dl & no medication; pre-diabetes: FPG 100-126 mg/dl and no medication; type 2 diabetes; FPG ≥126 mg/dl or using medication. Obesity (BMI≥30 kg/m^2^). **Fig S4.** The annual incidence rates of all-cause mortality by diabesity phenotypes in men and women, separately. Normoglycemia: FPG<100 mg/dl & no medication; pre-diabetes: FPG 100-126 mg/dl and no medication; type 2 diabetes; FPG ≥126 mg/dl or using medication. General obesity (BMI≥30 kg/m^2^).

## Data Availability

The datasets used and/or analyzed during the current study are available from the corresponding author upon reasonable request.

## References

[1] Tancredi M, Rosengren A, Svensson A-M, Kosiborod M, Pivodic A, Gudbjörnsdottir S (2015). Excess mortality among persons with type 2 diabetes. N Engl J Med.

[2] WHO (2018). The top 10 causes of death.

[3] IDF Diabetes Atlas 2021 – 10th edn. https://diabetesatlas.org/idfawp/resource-files/2021/07/IDF_Atlas_10th_Edition_2021.pdf. 35914061

[4] Riaz H, Khan MS, Siddiqi TJ, Usman MS, Shah N, Goyal A (2018). Association between obesity and cardiovascular outcomes: a systematic review and meta-analysis of Mendelian randomization studies. JAMA Netw Open.

[5] Ford JH, Lage MJ, Boye KS, Bae JP, Terrell KA, Bunck MC (2023). Five-year morbidity and mortality rates in a US population with obesity with and without prediabetes. J Diabetes Complicat.

[6] Doehner W, Gerstein HC, Ried J, Jung H, Asbrand C, Hess S (2020). Obesity and weight loss are inversely related to mortality and cardiovascular outcome in prediabetes and type 2 diabetes: data from the ORIGIN trial. Eur Heart J.

[7] Keller K, Münzel T, Ostad MA (2018). Sex-specific differences in mortality and the obesity paradox of patients with myocardial infarction ages >70 y. Nutrition.

[8] Zahir SF, Griffin A, Veerman JL, Magliano DJ, Shaw JE, Cao KL (2019). Exploring the association between BMI and mortality in Australian women and men with and without diabetes: the AusDiab study. Diabetologia.

[9] Paulin A, Manikpurage HD, Després JP, Thériault S, Arsenault BJ (2023). Sex-specific impact of body weight on atherosclerotic cardiovascular disease incidence in individuals with and without ideal cardiovascular health. J Am Heart Assoc.

[10] Hong S, Lee JH, Kim KM, Lee JW, Youn YJ, Ahn MS (2018). Is there a sex-related difference in the obesity paradox in systolic heart failure? Sex-related difference in the obesity paradox. Yonsei Med J.

[11] Ng AC, Delgado V, Borlaug BA, Bax JJ (2021). Diabesity: the combined burden of obesity and diabetes on heart disease and the role of imaging. Nat Rev Cardiol.

[12] Lim RBT, Chen C, Naidoo N, Gay G, Tang WE, Seah D (2015). Anthropometrics indices of obesity, and all-cause and cardiovascular disease-related mortality, in an Asian cohort with type 2 diabetes mellitus. Diabetes Metab.

[13] Song R, Chen X, He K, Hu X, Bai K, Shi W (2022). Associations of BMI with all-cause mortality in normoglycemia, impaired fasting glucose and type 2 diabetes mellitus among an elderly Chinese population: a cohort study. BMC Geriatr.

[14] Sims EA, Danforth Jr E, Horton ES, Bray GA, Glennon J, Salans L. Endocrine and metabolic effects of experimental obesity in man. In: Proceedings of the 1972 Laurentian Hormone Conference; 1973: Elsevier. 10.1016/b978-0-12-571129-6.50016-6 4750591

[15] Zhao Y, Liu Y, Sun H, Sun X, Yin Z, Li H (2018). Body mass index and risk of all-cause mortality with normoglycemia, impaired fasting glucose and prevalent diabetes: results from the Rural Chinese Cohort Study. J Epidemiol Community Health.

[16] Kong L, Qi Y, Ye C, Wang Y, Zhao Z, Li M (2022). Diabesity phenotype and the risks of cardiovascular disease and subclinical atherosclerosis: a prospective cohort study. Obesity.

[17] Azizi F, Zadeh-Vakili A, Takyar M (2018). Review of rationale, design, and initial findings: Tehran Lipid and Glucose Study. Int J Endocrinol Metab.

[18] Azizi F, Ghanbarian A, Momenan AA, Hadaegh F, Mirmiran P, Hedayati M (2009). Prevention of non-communicable disease in a population in nutrition transition: Tehran Lipid and Glucose Study phase II. Trials.

[19] Bild DE, Bluemke DA, Burke GL, Detrano R, Diez Roux AV, Folsom AR (2002). Multi-ethnic study of atherosclerosis: objectives and design. Am J Epidemiol.

[20] Investigators A (1989). The atherosclerosis risk in communit (ARIC) study: design and objectives. Am J Epidemiol.

[21] Dharod A, Soliman EZ, Dawood F, Chen H, Shea S, Nazarian S (2016). Association of asymptomatic bradycardia with incident cardiovascular disease and mortality: the multi-ethnic study of atherosclerosis (MESA). JAMA Intern Med.

[22] Sardarinia M, Akbarpour S, Lotfaliany M, Bagherzadeh-Khiabani F, Bozorgmanesh M, Sheikholeslami F (2016). Risk factors for incidence of cardiovascular diseases and all-cause mortality in a middle eastern population over a decade follow-up: Tehran lipid and glucose study. PLoS ONE.

[23] White AD, Folsom AR, Chambless LE, Sharret AR, Yang K, Conwill D (1996). Community surveillance of coronary heart disease in the Atherosclerosis Risk in Communities (ARIC) Study: methods and initial two years' experience. J Clin Epidemiol.

[24] Folsom AR, Yatsuya H, Nettleton JA, Lutsey PL, Cushman M, Rosamond WD (2011). Community prevalence of ideal cardiovascular health, by the American Heart Association definition, and relationship with cardiovascular disease incidence. J Am Coll Cardiol.

[25] Wright JD, Folsom AR, Coresh J, Sharrett AR, Couper D, Wagenknecht LE (2021). The ARIC (atherosclerosis risk in communities) study: JACC focus seminar 3/8. J Am Coll Cardiol.

[26] Alberti KG, Eckel RH, Grundy SM, Zimmet PZ, Cleeman JI, Donato KA (2009). Harmonizing the metabolic syndrome: a joint interim statement of the international diabetes federation task force on epidemiology and prevention; national heart, lung, and blood institute; American heart association; world heart federation; international atherosclerosis society; and international association for the study of obesity. Circulation.

[27] Khalili D, Azizi F, Asgari S, Zadeh-Vakili A, Momenan AA, Ghanbarian A (2018). Outcomes of a longitudinal population-based cohort study and pragmatic community trial: findings from 20 years of the Tehran Lipid and Glucose Study. Int J Endocrinol Metab.

[28] Magnussen C, Ojeda FM, Leong DP, Alegre-Diaz J, Amouyel P, Aviles-Santa L (2023). Global effect of modifiable risk factors on cardiovascular disease and mortality. New Engl J Med.

[29] Prentice RL (1986). A case-cohort design for epidemiologic cohort studies and disease prevention trials. Biometrika.

[30] Dam V, Van Der Schouw YT, Onland-Moret NC, Groenwold RH, Peters SA, Burgess S (2019). Association of menopausal characteristics and risk of coronary heart disease: a pan-European case–cohort analysis. Int J Epidemiol.

[31] Dwivedi AK, Dubey P, Cistola DP, Reddy SY (2020). Association between obesity and cardiovascular outcomes: updated evidence from meta-analysis studies. Curr Cardiol Rep.

[32] Brown OI, Drozd M, McGowan H, Giannoudi M, Conning-Rowland M, Gierula J (2023). Relationship among diabetes, obesity, and cardiovascular disease phenotypes: a UK Biobank Cohort Study. Diabetes Care.

[33] Cai X, Zhang Y, Li M, Wu JH, Mai L, Li J (2020). Association between prediabetes and risk of all cause mortality and cardiovascular disease: updated meta-analysis. BMJ.

[34] Sone H, Tanaka S, Iimuro S, Oida K, Yamasaki Y, Ishibashi S (2009). Waist circumference as a cardiovascular and metabolic risk in Japanese patients with type 2 diabetes. Obesity.

[35] Liu X-m, Liu Y-j, Zhan J, He Q-Q (2015). Overweight, obesity and risk of all-cause and cardiovascular mortality in patients with type 2 diabetes mellitus: a dose–response meta-analysis of prospective cohort studies. Eur J Epidemiol.

[36] Zhao Y, Qie R, Han M, Huang S, Wu X, Zhang Y (2021). Association of BMI with cardiovascular disease incidence and mortality in patients with type 2 diabetes mellitus: a systematic review and dose–response meta-analysis of cohort studies. Nutr Metab Cardiovasc Dis.

[37] Min Y-I, Gao Y, Anugu P, Anugu A, Correa A (2021). Obesity and overall mortality: findings from the Jackson Heart Study. BMC Public Health.

[38] Xu H, Cupples LA, Stokes A, Liu C-T (2018). Association of obesity with mortality over 24 years of weight history: findings from the Framingham Heart Study. JAMA Netw Open.

[39] Ward ZJ, Willett WC, Hu FB, Pacheco LS, Long MW, Gortmaker SL (2022). Excess mortality associated with elevated body weight in the USA by state and demographic subgroup: a modelling study. EClinicalMedicine.

[40] Lee EY, Lee Y-h, Yi S-W, Shin S-A, Yi J-J (2017). BMI and all-cause mortality in normoglycemia, impaired fasting glucose, newly diagnosed diabetes, and prevalent diabetes: a cohort study. Diabetes Care.

[41] Zahir SF, Griffin A, Veerman JL, Magliano DJ, Shaw JE, Cao K-AL (2019). Exploring the association between BMI and mortality in Australian women and men with and without diabetes: the AusDiab study. Diabetologia.

[42] Look ARG, Wing RR, Bolin P, Brancati FL, Bray GA, Clark JM (2013). Cardiovascular effects of intensive lifestyle intervention in type 2 diabetes. N Engl J Med.

[43] Look AHEAD Research Group (2016). Association of the magnitude of weight loss and changes in physical fitness with long-term cardiovascular disease outcomes in overweight or obese people with type 2 diabetes: a post-hoc analysis of the Look AHEAD randomised clinical trial. Lancet Diabetes Endocrinol.

[44] Batsis JA, Apolzan JW (2021). A systematic review of dietary supplements and alternative therapies for weight loss. Obesity.

[45] Kahan S, Fujioka K (2017). Obesity pharmacotherapy in patients with type 2 diabetes. Diabetes Spectr.

[46] Hadaegh F, Zabetian A, Sarbakhsh P, Khalili D, James W, Azizi F (2009). Appropriate cutoff values of anthropometric variables to predict cardiovascular outcomes: 7.6 years follow-up in an Iranian population. Int J Obes.

