# Supplementary material for: The impact of obesity on different glucose tolerance status with incident cardiovascular disease and mortality events over 15 years of follow-up: a pooled cohort analysis
Source: Diabetol Metab Syndr. 2024 Jan 25;16:27. doi: 10.1186/s13098-023-01253-0 (PMC10809520; doi:10.1186/s13098-023-01253-0)
Supplement: Supplementary file 1 — Additional file 1: Table S1. Baseline characteristics of subjects, stratified by study and gender. Fig S1. Study Flowchart CV Cardiovascular, ARIC Atherosclerosis Risk in Communities, MESA Multi-Ethnic Study of Atherosclerosis, TLGS Tehran Lipid and Glucose Study. Fig S2. The annual incidence rates of cardiovascular disease (CVD) by diabesity phenotypes in men and women, separately. Normoglycemia: FPG<100 mg/dl & no medication; pre-diabetes: FPG 100-126 mg/dl and no medication; type 2 diabetes; FPG ≥126 mg/dl or using medication. Obesity (BMI≥30 kg/m2). Fig S3. The annual incidence rates of cardiovascular (CV) mortality by diabesity phenotypes in men and women, separately. Normoglycemia: FPG<100 mg/dl & no medication; pre-diabetes: FPG 100-126 mg/dl and no medication; type 2 diabetes; FPG ≥126 mg/dl or using medication. Obesity (BMI≥30 kg/m2). Fig S4. The annual incidence rates of all-cause mortality by diabesity phenotypes in men and women, separately. Normoglycemia: FPG<100 mg/dl & no medication; pre-diabetes: FPG 100-126 mg/dl and no medication; type 2 diabetes; FPG ≥126 mg/dl or using medication. General obesity (BMI≥30 kg/m2). [file 13098_2023_1253_MOESM1_ESM.docx]

| **List of supplementary files** | |
| --- | --- |
| **File 1** | Study objective and design of cohorts |
| **Table S1.** | Baseline characteristics of subjects, stratified by study and gender |
| **Fig S1.** | Study Flowchart |
| **Fig S2.** | The annual incidence rates of cardiovascular disease (CVD) by diabesity phenotypes in men and women, separately |
| **Fig S3.** | The annual incidence rates of cardiovascular (CV)mortality by diabesity phenotypes in men and women, separately |
| **Fig S4.** | The annual incidence rates of all-cause mortality by diabesity phenotypes in men and women, separately |

**File 1.Study objective and design of cohorts**

**Atherosclerosis Risk in Communities Study (ARIC):** The ARIC study aims to investigate the underlying causes of atherosclerosis, a condition characterized by the buildup of plaque in the arteries, as well as its clinical outcomes. It also examines changes in cardiovascular risk factors, healthcare, and disease patterns in relation to race, gender, location, and time. The Cohort Component of ARIC was initiated in 1987, and four field centers located in Washington County, MD; Forsyth County, NC; Jackson, MS; and Minneapolis, MN were involved in randomly selecting and recruiting a sample of approximately 4,000 individuals aged 45 to 64 from specific populations in their respective communities. A total of 15,792 participants underwent thorough examinations, including the collection of medical, social, and demographic information. These participants were reevaluated every three years, with the first screening (baseline) taking place between 1987 and 1989, the second between 1990 and 1992, the third between 1993 and 1995, the fourth between 1996 and 1998, and the fifth time between 2011 and 2013 ([1](#_ENREF_1)). (More details are available at <https://sites.cscc.unc.edu/aric/description>)

**Multi-Ethnic Study of Atherosclerosis (MESA):** The MESA (Multi-Ethnic Study of Atherosclerosis) is a scientific investigation that focuses on studying the characteristics of cardiovascular disease that is not yet clinically evident (disease that has been detected through non-invasive methods before showing signs and symptoms). The purpose of the study is to understand the risk factors that can predict the progression of subclinical cardiovascular disease into clinically apparent disease, as well as the progression of subclinical disease itself. The study was initiated in the year 2000. Researchers involved in the MESA study examine a diverse group of individuals who are representative of the general population. The sample consists of 6,814 men and women between the ages of 45 and 84. These participants are asymptomatic, meaning they do not show any symptoms of cardiovascular disease. The study is conducted at six different field centers located in various regions across the United States. These centers are Johns Hopkins University in Baltimore, MD; Northwestern University in Chicago, IL; Wake Forest University in Forsyth County, NC; the University of California at Los Angeles in Los Angeles County, CA; Columbia University in Northern Manhattan and Southern Bronx, NY; and the University of Minnesota in St. Paul, MN. The participants in the study are ethnically diverse, with approximately 38 percent of them being white, 28 percent African American, 22 percent Hispanic, and 12 percent Asian, primarily of Chinese descent.The initial examination phase of the study began in July 2000 and spanned over a period of 24 months. This first examination was designed to be the most comprehensive, covering various aspects related to cardiovascular health. Since 2000, a total of six examinations have been conducted as part of the study.Throughout the study, participants were regularly contacted every 9 to 12 months to evaluate their clinical morbidity (disease incidence) and mortality (death rates). The final 18 months of the study were dedicated to closing out the research and analyzing the collected data, which will be used for publication and further scientific analysis. ([2](#_ENREF_2)). (More details are available at https://www.mesa-nhlbi.org/aboutMESA.aspx)

**Tehran Lipid and Glucose Study (TLGS):** The Tehran Lipid and Glucose Study (TLGS) is a scientific research project that involves studying a population of people aged three years and above who live in the urban area of Tehran, the capital of Iran. The main goal of this study is to determine how common and how often non-communicable diseases occur, as well as identify the factors that contribute to these diseases. Additionally, the study focuses on developing strategies for promoting a healthy lifestyle to counteract these risk factors. The enrollment process for the TLGS took place in two stages: the first stage occurred from January 31, 1999, to July 03, 2001, and the second stage took place from October 20, 2001, to September 22, 2005. Data collection will continue for a minimum of 20 years, with intervals of approximately three years between each phase (phase III: 2005–2008, phase IV: 2008–2011, phase V: 2011–2014, and phase VI: 2015–2018)([3](#_ENREF_3)).

| Table S1. Baseline characteristics of subjects, stratified by study and gender | | | | | | | | |
| --- | --- | --- | --- | --- | --- | --- | --- | --- |
|  | **Men** | | | | **women** | | | |
| Variables | **All**  **(n=8257)** | **ARIC**  **(n=4443)** | **MESA**  **(n=1640)** | **TLGS**  **(n=2174)** | **All**  **(n=9927)** | **ARIC**  **(n=5549)** | **MESA**  **(n=1436)** | **TLGS**  **(n=2942)** |
| Age (years) | 50.2( 7.1) | 52.5 (4.6) | 52.6 (4.5) | 43.7 (8.6) | 49.7 (7.1) | 52.0 (4.6) | 52.5 (4.5) | 44.0 (8.6) |
| SBP (mmHg) | 119.2 (17.4) | 120.7 (17.3) | 118.3 (19.7) | 116.8 (15.6) | 117.3 (18.2) | 118.0 (18.8) | 120.1 (16.5) | 114.6(17.5) |
| DBP (mmHg) | 74.6 (11.2) | 75.6 (11.2) | 69.4 (10.3) | 76.5 (10.6) | 73.4 (10.5) | 71.9 (10.7) | 75.7(9.2) | 75.3 (10.2) |
| BMI (kg/m^2^) | 27.7 (4.8) | 27.6 (4.2) | 29.2 (6.6) | 26.8 (4.1) | 28.1 (5.5) | 27.6 (6.1) | 28.2(4.4) | 29.2 (4.8) |
| WC (cm) | 97.4 (12.4) | 99.3 (10.9) | 96.2 (16.9) | 94.5 (10.5) | 94.2 (14.3) | 94.3 (15.6) | 98.9 (12.2) | 91.9 (12.1) |
| Non-HDL-C (mg/dl) | 157.0(132.0-184.8) | 165.4(138.5-193.0) | 140.0(117.0-164.0) | 155.0(132.0-181.0) | 152.0(126.9-181.3) | 153.1(126.0-184.8) | 146.0(123.0-170.0) | 153.5(129.0-182.0) |
| FPG (mg/dl) | 96.0(88.6-105.0) | 101.0(95.0-109.0) | 85.0(80.0-94.0) | 91.0(86.0-99.0) | 94.0(87.6-103.0) | 97.0(91.0-105.0) | 89.0(83.0-98.0) | 90.0(84.0-99.0) |
| General obesity (yes) | 2132 (25.8) | 1058 (23.8) | 644 (39.3) | 430 (19.8) | 3204 (32.3) | 1586 (28.6) | 448 (31.2) | 1170 (39.8) |
| Education, years |  |  |  |  |  |  |  |  |
| <6 | 1252(15.2) | 855(19.2) | 12(0.7) | 385(17.7) | 2054(20.7) | 1037(18.7) | 3(0.2) | 1014(34.5) |
| 6-12 | 31.3(37.6) | 1680(37.8) | 120(7.3) | 1303(59.9) | 4294(43.3) | 2592(46.7) | 105(7.3) | 1597(54.3) |
| ≥12 | 3902(74.2) | 1908(42.9) | 1508(92.0) | 486(22.4) | 3579(36.0) | 1920(34.6) | 1328(92.5) | 331(11.2) |
| Current smoker(yes) | 2343 (28.4) | 1265 (28.5) | 271 (16.5) | 807 (37.1) | 1914 (19.3) | 1429 (25.7) | 291 (20.3) | 194 (6.5) |
| Hypertension (yes) | 2348 (28.5) | 1475 (33.2) | 547 (33.4) | 326 (15.0) | 2981 (30.0) | 2038 (36.7) | 446 (31.1) | 497 (16.9) |
| Diabetes status |  |  |  |  |  |  |  |  |
| Normoglycemia | 4975(60.2) | 1984(44.6) | 1347(82.1) | 1644(75.6) | 6682(67.3) | 3372(60.8) | 1099(76.5) | 2211(75.2) |
| Pre-diabetes | 2564(31.1) | 2048(46.1) | 150(9.1) | 366(16.8) | 2329(23.5) | 1696(30.6) | 184(12.8) | 449(15.3) |
| Type 2 diabetes | 718(8.7) | 411(9.2) | 143(8.7) | 164(7.5) | 916(9.2) | 481(8.7) | 153(10.6) | 282(9.6) |
| FHCVD (yes) | 1471 (17.8) | 446 (10.0) | 856 (52.2) | 169 (7.8) | 1455 (14.7) | 643 (11.6) | 679 (47.3) | 133 (4.5) |
| Prevalent CVD (yes) | 506 (6.2) | 391 (9.9) | - | 115 (5.3) | 245 (2.5) | 154 (2.8) | - | 91 (3.1) |
| Continuous variables are shown as mean (standard deviation; SD) for normal distributed and median (interquartile range: IQR) for skewed variable (e.g FPG, non-HDL-C) and categorical variables are presented as number (%).  ARIC: Atherosclerosis Risk in Communities; MESA: Multi-Ethnic Study of Atherosclerosis; TLGS: Tehran Lipid and Glucose Study; SBP: systolic blood pressure; DBP: diastolic blood pressure; BMI: body mass index; WC: waist circumference; FPG: fasting plasma glucose; non-HDL-C: non-high density lipoprotein cholesterol; FHCVD: family history cardiovascular disease; CVD: cardiovascular disease. | | | | | | | | |

| 20882 individuals aged 30-60 years from three population based study:  TLGS (2002-2005): 5560  ARIC (1987-1989): 12162  MESA (2000-2002): 3100  18184 Study population from three cohorts  (9927 women)  17433 Study population from three cohorts  (9682 women)  CV and all-cause Mortality related events  Exclusion:  Missing data on baseline covariates: 2594  Loss to follow-up: 44  CVD events  Exclusion:  Missing data on baseline covariates: 2594  Loss to follow-up: 44  Exclusion:  Prevalence CVD: 942  Missing data on baseline covariates: 2467  Loss to follow-up: 40  Exclusion:  Missing data on baseline covariates: 2654  Loss to follow-up: 44 |
| --- |
| **Fig S1**. Study Flowchart  CV: Cardiovascular; ARIC: Atherosclerosis Risk in Communities; MESA: Multi-Ethnic Study of Atherosclerosis; TLGS: Tehran Lipid and Glucose Study |

|  |
| --- |
| **Fig S2.** The annual incidence rates of cardiovascular disease (CVD) by diabesity phenotypes in men and women, separately  Normoglycemia: FPG<100 mg/dl & no medication; pre-diabetes: FPG 100-126 mg/dl and no medication; type 2 diabetes; FPG ≥126 mg/dl or using medication.  Obesity (BMI≥30 kg/m^2^). |

|  |
| --- |
| **Fig S3.** The annual incidence rates of cardiovascular (CV)mortality by diabesity phenotypes in men and women, separately  Normoglycemia: FPG<100 mg/dl & no medication; pre-diabetes: FPG 100-126 mg/dl and no medication; type 2 diabetes; FPG ≥126 mg/dl or using medication.  Obesity (BMI≥30 kg/m^2^). |

|  |
| --- |
| **Fig S4.** The annual incidence rates of all-cause mortality by diabesity phenotypes in men and women, separately  Normoglycemia: FPG<100 mg/dl & no medication; pre-diabetes: FPG 100-126 mg/dl and no medication; type 2 diabetes; FPG ≥126 mg/dl or using medication.  General obesity (BMI≥30 kg/m^2^). |

**References**

1. Investigators A. The Atherosclerosis risk in COMMUNIT (ARIC) study: design and objectives. American journal of epidemiology. 1989;129(4):687-702.

2. Bild DE, Bluemke DA, Burke GL, Detrano R, Diez Roux AV, Folsom AR, et al. Multi-ethnic study of atherosclerosis: objectives and design. American journal of epidemiology. 2002;156(9):871-81.

3. Azizi F, Zadeh-Vakili A, Takyar M. Review of rationale, design, and initial findings: Tehran Lipid and Glucose Study. International journal of endocrinology and metabolism. 2018;16(4 Suppl).
